# Antibody-based soluble and membrane-bound TWEAK mimicking agonists with FcγR-independent activity

**DOI:** 10.3389/fimmu.2023.1194610

**Published:** 2023-07-11

**Authors:** Olena Zaitseva, Annett Hoffmann, Margaretha Löst, Mohamed A. Anany, Tengyu Zhang, Kirstin Kucka, Armin Wiegering, Christoph Otto, Harald Wajant

**Affiliations:** ^1^ Division of Molecular Internal Medicine, Department of Internal Medicine II, University Hospital Würzburg, Würzburg, Germany; ^2^ Department of General, Visceral, Transplant, Vascular and Pediatric Surgery, University Hospital Würzburg, Würzburg, Germany; ^3^ Department of Microbial Biotechnology, Institute of Biotechnology, National Research Center, Giza, Egypt

**Keywords:** agonistic antibodies, cell death, FcγR, Fn14, NFκB, TNF receptor superfamily, TWEAK

## Abstract

Fibroblast growth factor (FGF)-inducible 14 (Fn14) activates the classical and alternative NFκB (nuclear factor ‘kappa-light-chain-enhancer’ of activated B-cells) signaling pathway but also enhances tumor necrosis factor (TNF)-induced cell death. Fn14 expression is upregulated in non-hematopoietic cells during tissue injury and is also often highly expressed in solid cancers. In view of the latter, there were and are considerable preclinical efforts to target Fn14 for tumor therapy, either by exploiting Fn14 as a target for antibodies with cytotoxic activity (e.g. antibody-dependent cellular cytotoxicity (ADCC)-inducing IgG variants, antibody drug conjugates) or by blocking antibodies with the aim to interfere with protumoral Fn14 activities. Noteworthy, there are yet no attempts to target Fn14 with agonistic Fc effector function silenced antibodies to unleash the proinflammatory and cell death-enhancing activities of this receptor for tumor therapy. This is certainly not at least due to the fact that anti-Fn14 antibodies only act as effective agonists when they are presented bound to Fcγ receptors (FcγR). Thus, there are so far no antibodies that robustly and selectively engage Fn14 signaling without triggering unwanted FcγR-mediated activities. In this study, we investigated a panel of variants of the anti-Fn14 antibody 18D1 of different valencies and domain architectures with respect to their inherent FcγR-independent ability to trigger Fn14-associated signaling pathways. In contrast to conventional 18D1, the majority of 18D1 antibody variants with four or more Fn14 binding sites displayed a strong ability to trigger the alternative NFκB pathway and to enhance TNF-induced cell death and therefore resemble in their activity soluble (TNF)-like weak inducer of apoptosis (TWEAK), one form of the natural occurring ligand of Fn14. Noteworthy, activation of the classical NFκB pathway, which naturally is predominately triggered by membrane-bound TWEAK but not soluble TWEAK, was preferentially observed with a subset of constructs containing Fn14 binding sites at opposing sites of the IgG scaffold, e.g. IgG1-scFv fusion proteins. A superior ability of IgG1-scFv fusion proteins to trigger classical NFκB signaling was also observed with the anti-Fn14 antibody PDL192 suggesting that we identified generic structures for Fn14 antibody variants mimicking soluble and membrane-bound TWEAK.

## Introduction

Fibroblast growth factor (FGF)-inducible 14 (Fn14) is an unusual small member of the tumor necrosis factor (TNF) receptor superfamily (TNFRSF) with an extracellular domain only comprising a single cysteine rich domain, and an intracellular tail of 28 amino acids which contains a binding site for proteins of the TNF receptor associated factor (TRAF) family (1, 2). Fn14 is dynamically and highly expressed during development but in healthy adult organisms Fn14 expression is largely limited to heart, ovary and mesenchymal progenitor cells (1, 3). Fn14 expression is, however, strongly upregulated in non-hematopoietic cells after tissue injury irrespective of the underlying reason (4). Since tumor development is inevitably associated with tissue damage and tissue remodeling, Fn14 expression is also often high in tumor cells of non-hematopoietic origin and non-transformed non-hematopoietic cells of the tumor microenvironment (4, 5). The expression of Fn14 can be therefore considered as a bona fide marker for tissue remodeling and tissue injury. Fn14 signal transduction can be triggered by tumor necrosis factor (TNF)-like weak inducer of apoptosis (TWEAK), a ligand of the TNF superfamily (TNFSF) which occurs in two forms, namely as transmembrane TWEAK (memTWEAK) and as soluble TWEAK (sTWEAK) which is released from memTWEAK by proteolytic processing (6). Similar to other ligands of the TNFSF, memTWEAK and sTWEAK form homotrimeric molecules which can bind three receptor molecules (4). TWEAK expression has been shown for a variety of cell lines and cell types by immunohistochemistry and RT-PCR, but memTWEAK expression has doubtless only be demonstrated on monocytes, dendritic cells and natural killer (NK) cells and a very few tumor cell lines (4).

Importantly, sTWEAK and memTWEAK trigger different states of Fn14 activity. In response to sTWEAK Fn14 efficiently stimulates the alternative NFκB signaling pathway and sensitizes for TNF-induced cell death (4). Transmembrane TWEAK triggers the same Fn14 signaling events as sTWEAK but in addition enables Fn14 to activate also the classical NFκB pathway (4). Manifold and complex functions of the TWEAK/Fn14 system have been described in tissue repair and regeneration. For example, it has been demonstrated that the TWEAK/Fn14 system promotes regenerative responses after the injury of muscles, pancreas and the liver (7–9). However, excessive and/or chronic engagement of the TWEAK/Fn14 system can also result in tissue repair-associated adverse effects, such as fibrosis and inflammation (10–12). Thus, dependent on the context and the disease considered, both the inhibition but also the stimulation of Fn14 can elicit beneficial therapeutic effects (4).

The inhibition of the TWEAK/Fn14 system can be straightforwardly achieved by help of soluble Fn14-Fc fusion proteins, TWEAK neutralizing antibodies or blocking, effector function-dead Fn14 antibody variants (4). Specific stimulation of Fn14 signaling is, however, more challenging. Conventional sTWEAK has an extremely low serum half-life (< 20 min) (13) and oligomeric sTWEAK variants, which display memTWEAK-like activity, are modestly produced and are more challenging in translational development than antibodies. The reagents of choice to stimulate Fn14 *in vivo* are therefore agonistic antibodies but here arises two fundamental problems: First, although some anti-Fn14 IgG antibodies can promote to some extend in certain cell lines p100 processing, a hallmark of the alternative NFκB pathway, they are largely not agonistic and require anchoring to Fcγ receptors or oligomerization, e.g. by protein G or antibody crosslinking, to become fully and strongly agonistic (14–16). Antibody oligomerization by protein G or secondary antibodies, however, is no practicable translational option and the FcγR-binding dependent mode of anti-Fn14 agonism is inevitably associated with triggering FcγR effector function what can disturb the anticipated therapeutic effect. Second, if Fn14 antibodies become agonistic by the aforementioned means they mimic memTWEAK, thus mimicry of sTWEAK seems hardly possible with Fn14 antibodies.

Here, we analyzed a variety of tetra-, hexa- and octavalent antibody variants composed of Fn14-specific Fab- and scFv domains with respect to their Fn14 agonism. All the Fab-scFv chimeric multivalent anti-Fn14 antibody variants showed inherent and partly strong memTWEAK-mimicking agonism. Surprisingly, multivalent “scFv” domain-only variants preferentially mimicked sTWEAK activity on Fn14. In sum, these novel potent antibody-based Fn14 agonists with FcγR-independent activity offer an alternative to recombinant TWEAK molecules to evaluate the clinical potential of pure Fn14 agonism *in vivo*.

## Results

### Construction of tetra-, hexa- and octavalent anti-Fn14 variants

**Figure 1:**
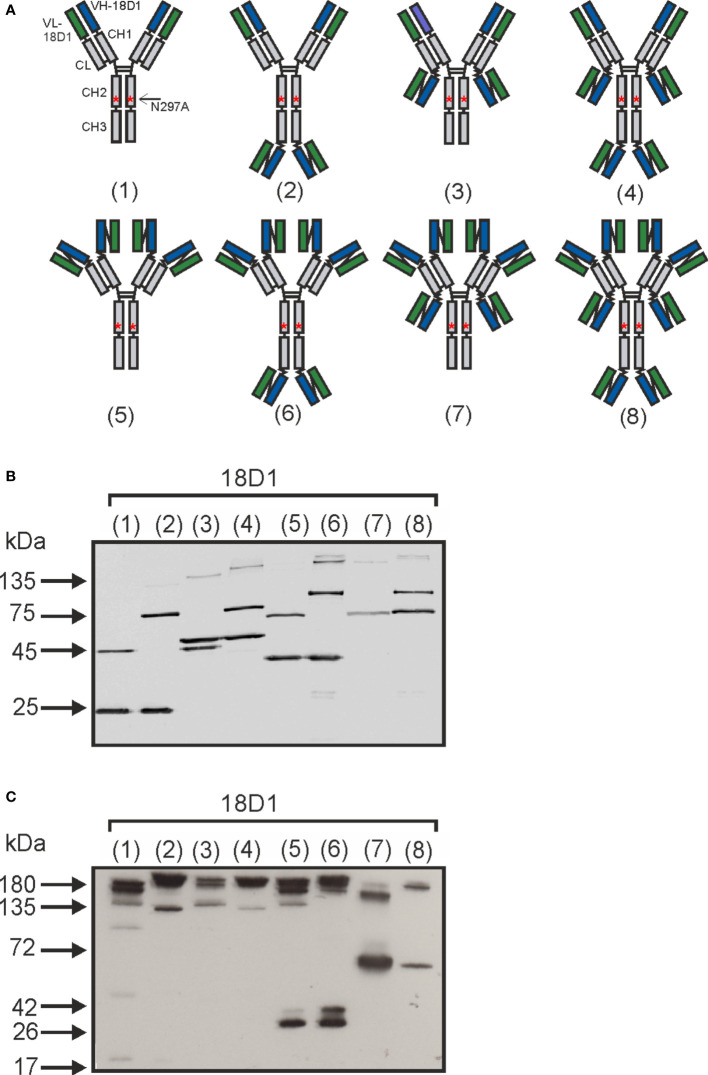
Antibody variants of the anti-Fn14-antibody 18D1. **(A)** Scheme of the domain architecture of the various 18D1 variants used in this study. **(B, C)** Western blot analysis of 10 µl supernatant of HEK293 cells transiently expressing the proteins shown in **(A)**. Samples in **(C)** were dissolved in Laemmli buffer without β-mercaptoethanol, thus without reduction.

In view of the fact that anti-Fn14 antibodies can acquire memTWEAK-like activity upon crosslinking (15), we generated various multivalent variants of the anti-Fn14 antibody 18D1 (14) and analyzed the ability of these molecules to stimulate Fn14 *in vitro*. To obtain tetravalent 18D1 variants, we genetically fused a scFv domain derived of 18D1 to the C-terminus of the heavy chain (HC) or light chain (LC) of 18D1-IgG1(N297A) (**Figure 1A**, construct 18D1-(1)), a 18D1-IgG1 variant with a point mutation destroying/reducing FcγR binding, resulting in the constructs 18D1-(2) and 18D1-(3) shown in **Figure 1A**. Alternatively, to have a tetravalent variant with four similarly oriented Fn14 binding sites on the same side of the antibody scaffold, we replaced the variable domains of the heavy (VH) and light chain (VL) of the parental 18D1-IgG1 antibody by scFv:18D1 domains (**Figure 1A**, construct 18D1-(5)). Hexameric 18D1 variants were furthermore generated by fusing the scFv:18D1 domain to the C-termini of the heavy and the light chain of 18D1-(1) (**Figure 1A**, construct 18D1-(4)) and by fusing this domain to the C-terminus of the heavy or light chain of construct 18D1-(5) (**Figure 1A**, constructs 18D1-(6) and 18D1-(7)). Finally, an octameric variant was obtained by fusing the scFv:18D1 domain to the C-terminus of both the heavy and light chain of construct 18D1-(5) (**Figure 1A**, construct 18D1-(8)). All antibody constructs were produced by transient co-transfection of HEK293 cells with expression plasmids encoding the corresponding Flag-tagged LC and HC variants. Productivity of the parental 18D1 antibody and all variants derived thereof was largely comparable (**Figures 1B, C**). Western blot analysis under non-reducing conditions suggested, furthermore, that constructs (1) to (4) undergo efficient disulfide bond dimerization while the scFv:18D1-”only” variants showed significant fractions of non-paired light chains (**Figure 1C**).

### Oligovalent 18D1 variants enhance TNF-induced toxicity and trigger the alternative NFκB pathway but substantially differ in their ability to induce IL8

**Figure 2:**
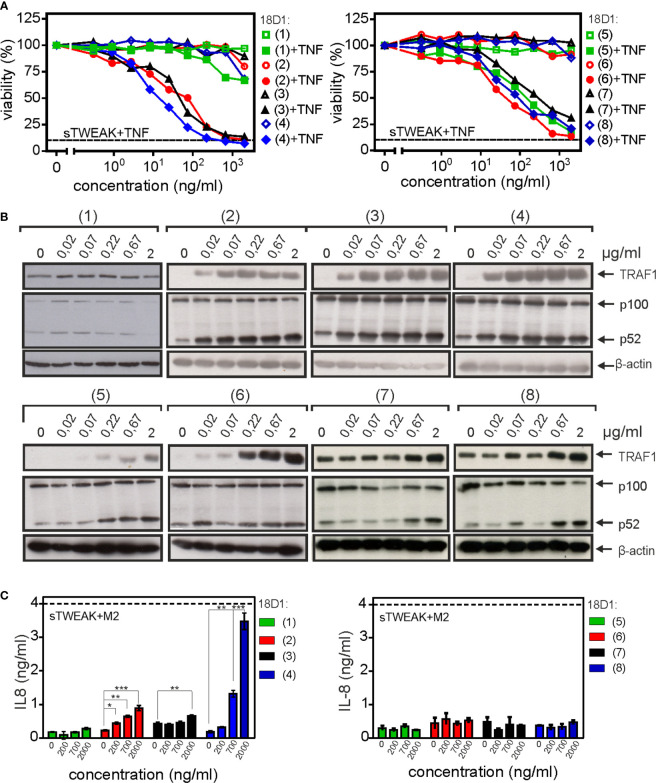
Triggering of Fn14 signaling by oligovalent 18D1 constructs. **(A)** HeLa-RIPK3-FADD_KO_ cells were treated with 1 ng/ml TNF which alone induces no or only modest cell death. Cells were treated in addition with cell culture supernatants containing the indicated 18D1 constructs. Cotreatment with (200 ng/ml) Flag-sTWEAK was performed to define maximum TNF killing in TWEAK-sensitized cells indicated by the dotted line. One representative experiment of three is shown. **(B)** HT1080 cells were treated overnight with cell culture supernatants containing the various 18D1 constructs or with Flag-sTWEAK. Total cell lysates were analyzed by western blotting with respect to p100 processing and expression of the alternative NFκB pathway target TRAF1. **(C)** HT1080 cells were treated with the various 18D1 constructs or with anti-Flag M2 oligomerized Flag-sTWEAK over night. IL8 concentrations were evaluate by ELISA. Data shown were averaged from three independent experiments and were analyzed by one way ANOVA and Bonferroni post test. *p < 0.05, **p < 0.01, ***p < 0.001.

**Figure 3:**
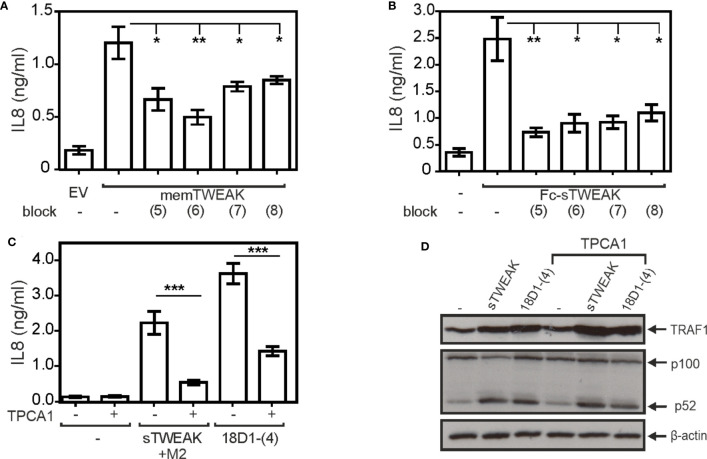
18D1-(5) to 18D1-(8) inhibit memTWEAK-induced classical NFκB signaling pathway-mediated IL8 expression. **(A, B)** HT1080 cells were pretreated with 30 µg/ml of the indicated 18D1 variants and were then challenged overnight with memTWEAK and, as a negative control, empty vector (EV) transfected HEK293 cells **(A)** or with 500 ng/ml Fc-sTWEAK **(B)**. Shown are the average of 7 **(A)** and 4 **(B)** independent experiments. Each construct type ((5), (6), (7) and (8)) was compared with the control using two-tailed t-test. *p < 0.05, **p < 0.01 **(C)** HT1080 cells were stimulated with anti-Flag antibody M2 (0.5 µg/ml) oligomerized Flag-sTWEAK (200 ng/ml) or 1 µg/ml 18D1-(4) in the presence and absence of the IKK2 inhibitor TPCA-1 (20 µM). Next day, IL8 production was again determined by ELISA. The effect of TPCA-1 on basal, sTWEAK/M2- and 18D1-(4)-induced IL8 production was evaluated in each case by the two-tailed t-test. ***p < 0.001. **(D)** HT1080 cells were stimulated with 200 ng/ml Flag-sTWEAK (200 ng/ml) or 1 µg/ml 18D1-(4) in the presence and absence of TPCA-1 (20 µM) and the next day, p100 processing and TRAF1 induction were assayed by western blotting.

With exception of the parental bivalent 18D1-(1) antibody variant, all anti-Fn14 constructs enhanced TNF-induced toxicity to a comparable extent as sTWEAK with ED50-values below 100 ng/ml (**Figure 2A**). All the oligovalent 18D1 constructs triggered furthermore robust p100 processing starting at concentrations of app. 20 ng/ml for 18D1-(2) to 18D1-(4) and of app. 200 ng/ml for 18D1-(5) to 18D1-(8). The conventional antibody variant (1), however, remained inactive in this respect and showed no p100 processing even at the highest concentrations of 2 µg/ml (**Figure 2B**). Similarly, with exception of 18D1-(1) all constructs upregulated TRAF1 expression which is controlled by the alternative NFκB pathway (**Figure 2B**). With respect to the induction of IL8, however, there was a clear difference between the constructs. IL8 is a prototypic target of the classical NFκB pathway and is accordingly not or only poorly induced by sTWEAK but efficiently by oligomerized sTWEAK and memTWEAK (17). Construct 18D1-(2) and especially construct 18D1-(4) displayed varying but significant and robust IL8 induction and reached in the case of the hexameric construct 18D1-(4) the maximum response that is induced by anti-Flag oligomerized Flag-sTWEAK (**Figure 2C**). In contrast, all 18D1-derived constructs with replacement of the VH and VL domains by the scFv:18D1 domain (18D1-(5) to 18D1-(8)) remained largely inactive (**Figure 2C**). Moreover, 18D1-(5) to 18D1-(8), despite their ability to trigger alternative NFκB signaling and enhancement of TNF-induced cell death (**Figures 2A, B**), inhibited IL8 induction by memTWEAK expressing transfectants and hexameric Fc-sTWEAK which has memTWEAK like activity (**Figures 3A, B**). In this respect, these constructs again resemble soluble TWEAK which also inhibits the memTWEAK-induced IL8 response (**Supplemental Data Figure S1**). The IKK2-specific inhibitor TPCA-1 efficiently inhibited IL8 induction by oligomerized sTWEAK and 18D1-(4) but showed no effect on p100 processing (**Figures 3C, D**) confirming that Fn14-mediated IL8 induction reflected activation of the classical NFκB pathway.

**Figure 4:**
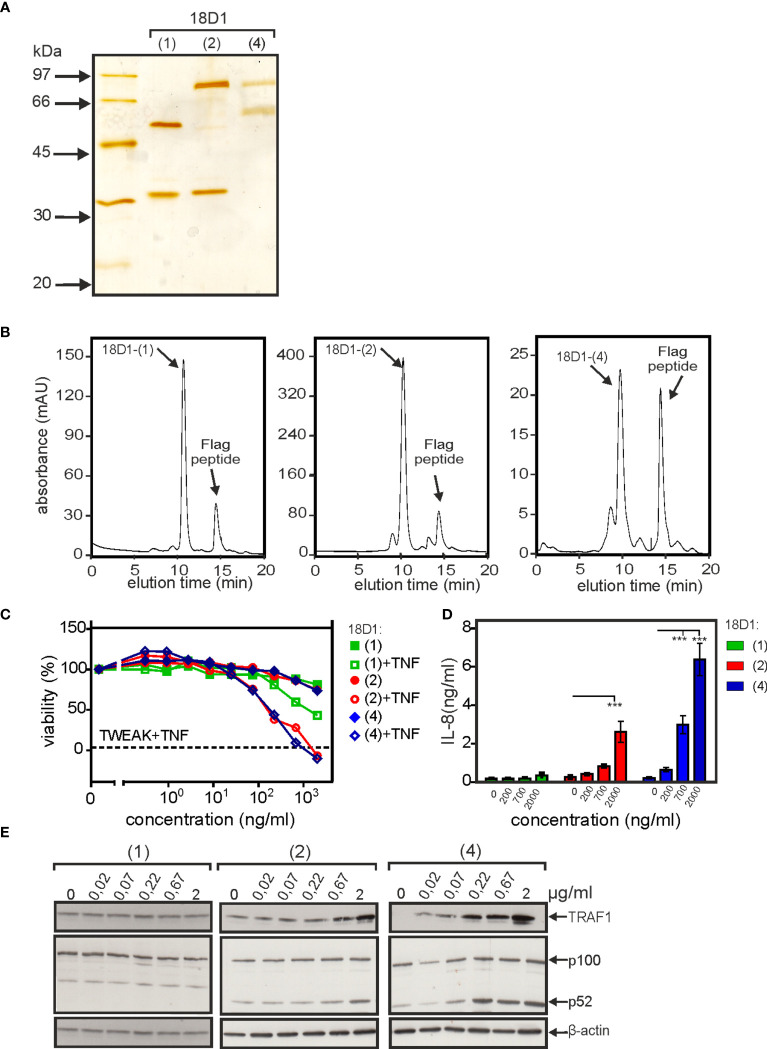
Purification and analysis of tetra- and hexavalent anti-Fn14 18D1 variants. **(A)** Purified proteins were separated by SDS-PAGE and visualized by silverstaining. **(B)** Gelfiltration analysis of purifed proteins. **(C–E)** Analysis of the ability of purified proteins from part B to trigger enhancement of TNF-induced toxicity in HeLa-RIPK3-FADD_KO_ cells **(C)** and IL8 production **(D)** and p100 processing with TRAF1 induction in HT1080 cells **(E)**. IL8 data **(D)** were averaged from seven independent experiments and were analyzed by one way ANOVA and Bonferroni post test. ***p < 0.001.

So far, we analyzed the activities of the various 18D1 variants by help of supernatants of cells producing these molecules. As mentioned above, the latent agonistic activity of anti-Fn14 antibodies can be unleashed by antibody crosslinking/oligomerizing reagents such as protein G (15). Therefore, to verify that the agonistic properties observed for the oligomeric 18D1 variants are not due to aggregation of the molecules and indeed mirrors intrinsic activity, we purified the parental antibody along with the prototypic variants 18D-(2) and 18D1-(4) and analyzed them by gel filtration (**Figures 4A, B**). All three proteins eluted in gel filtration largely as a single molecular species with MW well corresponding to Fc domain-dimerized molecules (**Figure 4B**). Functional analysis revealed furthermore that purification did not affect the functional properties of the molecules. The purified proteins 18D1-(2) and 18D1-(4) still enhanced TNF-induced cell death in HeLa-RIPK3-FADD_KO_ cells (**Figure 4C**) and classical (**Figure 4D**, **Supplemental Data Figure S2**) and alternative NFκB signaling (**Figure 4E**) in HT1080 cells.

### Format (2) and (4) variants of the anti-Fn14 mAb PDL192 also displays memTWEAK-like activity

**Figure 5:**
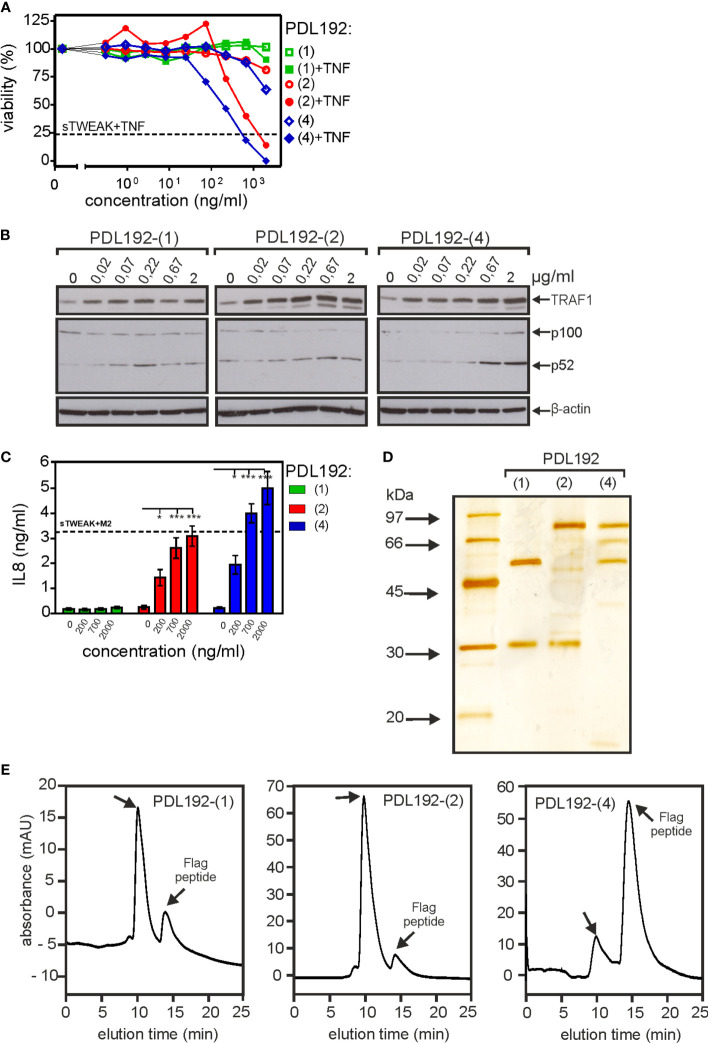
Triggering of Fn14 signaling by PDL192 type (2) and type (4) constructs. **(A-C)** The activity of PDL192-(2) and PDL192-(4) were analyzed as in **Figure 2** with respect to enhancement of TNF-induced cell death in HeLa-RIPK3-FADD_KO_ cells **(A)**, stimulation of p100 processing and TRAF1 indcution **(B)** and upregulation of IL8 production **(C)**. IL8 data were averaged from seven independent experiments and were analyzed by one way ANOVA and Bonferroni post test. *p < 0.05, ***p < 0.001. **(D, E)** Purified PDL192-(2) and PDL192-(4) along with the parental antibody PDL192 were analyzed by SDS-PAGE and silverstaining **(A)** or gelfiltration analysis **(E)**.

To proof that genetic fusion of Fn14-specific scFv domains to anti-Fn14 antibodies generally favors the realization of memTWEAK mimicking agonism, we generated and evaluated construct types (2) and (4) of a second anti-Fn14 antibodies, namely PDL192. Previous studies showed that 18D1 and PDL192 recognize different epitopes on Fn14 (14). Moreover, the two antibodies also differ in the ability to block TWEAK binding. While 18D1 inhibits TWEAK binding, PDL192 does not compete with ligand binding (14). As reported previously, the IgG1(N297A) version of PDL192 showed no agonism while its tetravalent and hexavalent derivatives, however, stimulated p100 processing and enhanced TNF-induced cell death (**Figures 5A, B**). Both constructs also elicited strong stimulation of IL8 induction (**Figure 5C**). These data suggest that type (2) and (4) antibody variants generally confer FcγR-independent memTWEAK-like agonism. To rule out again that the observed agonistic activity of the type (2) and (4) constructs of PDL192 did not result from unspecific aggregation, we purified both constructs by anti-Flag agarose affinity chromatography (**Figure 5D**). All PDL192 variants proteins were efficiently purified and showed almost no high molecular weight species (**Figure 5D**). Thus, the observed agonism of type (2) and type (4) anti-Fn14 variants is molecule intrinsic, too.

### Antitumoral activity of 18D1-(2)

**Figure 6:**
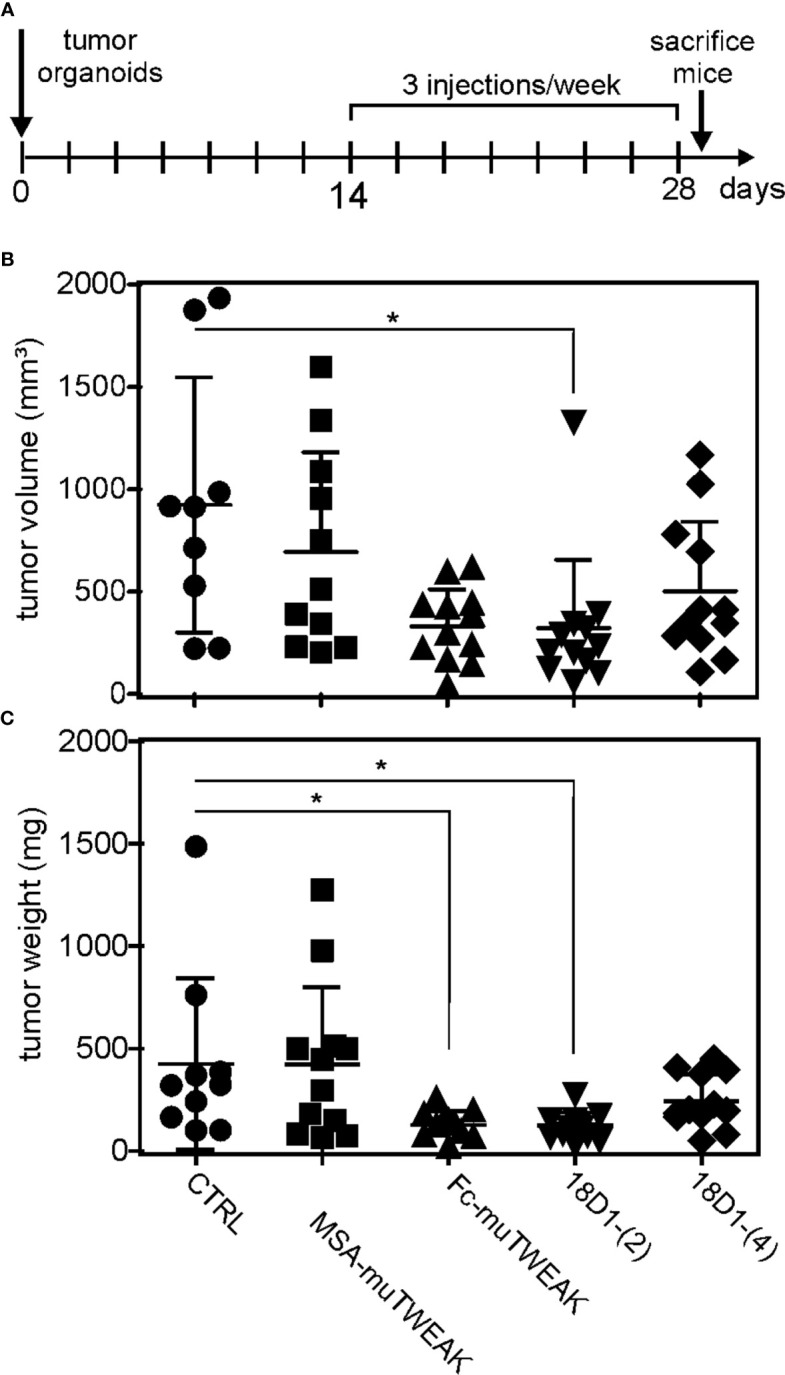
Fc-TWEAK and 18D1-(2) inhibit the growth of established murine tumor organoid-induced tumors. **(A)** Scheme of animal treatment. **(B, C)** Tumor weight and tumor volume of mice treated with fusion proteins of murine soluble TWEAK (muTWEAK) with murine serum albumin (MSA-muTWEAK) or human IgG1-derived Fc (Fc-muTWEAK), 18D1-(2) and 18D1-(4) were compared with the tumor weight and volume of control mice treated with NaCl physiological solution (CTRL) using the non parametric Kruska-Wallis test with Dunn’s multiple comparison post test. N = 9-12; *p < 0.05.

Fn14 expressing mouse tumor organoids (MTOs) (**Supplemental Data Figure S3**), derived from tumors originating from *Apc^ko/ko^, Kras^LSL-G12D^*, *Tgfbr2^ko/ko^* and *Trp53^ko/ko^* intestinal stem cells that imitate the microenvironment and severity of human colorectal cancer (18) were expanded *in vitro* and injected subcutaneously into the flank of syngeneic C57BL/6J recipients. Two weeks after organoid injections, when they developed into macroscopically established tumors, mice were treated three times per week for two weeks with 200 µg of 18D1-(2) or 18D1-(4) (**Figure 6A**). For comparison, mice were treated with MSA-muTWEAK, a fusion protein of murine sTWEAK with murine serum albumin (MSA) and thus prolonged serum retention, and Fc-muTWEAK, a hexameric murine sTWEAK variant with membrane TWEAK-like activity. Tumor weight and tumor mass were significantly reduced in mice treated with 18D1-(2) and there was also significantly reduced tumor mass after Fc-muTWEAK treatment (**Figures 6B, C**). MSA-muTWEAK and 18D1-(4) treated mice showed on average reduced tumor growth but this did not attain statistical significance (**Figures 6B, C**).

## Discussion

Against the background that Fn14 is regularly expressed at high levels on tumor cells of non-hematopoietic origin but also on various cells of the tumor microenvironment (4, 5), it is considered as a promising target for cancer therapy. In order to use the tumor-associated Fn14 expression therapeutically, three different approaches can be considered.

First, killing of Fn14^+^ tumor cells with anti-Fn14 antibodies with natural cytotoxic effector activity, such as ADCC (antibody-dependent cellular cytotoxicity), ADCP (antibody-dependent cellular phagocytosis) and CDC (complement-dependent cytotoxicity) or an artificial cytotoxic payload, such as a conjugated antitumor drug. Indeed, antitumoral efficacy has been demonstrated in preclinical *in vivo* models with the ADCC-inducing anti-Fn14 antibodies PDL192/Enavatuzumab and P4A8/BIIB036 (19–22) but also with a 18D1 version with enhanced ADCC activity (14). Noteworthy, there is evidence that the antitumoral effects of PDL192/Enavatuzumab and P4A8/BIIB036 were not only based on ADCC but also involve Fn14-mediated NFκB activation although *in vitro* these antibodies have no (or only very low) agonistic activity or even act as antagonists (14, 15, 19, 20, 22). Thus, triggering of Fn14 signaling by FcγR-bound antibody molecules may have contributed to the antitumoral effects reported for PDL192/Enavatuzumab and P4A8/BIIB036 *in vivo*. Antitumoral efficacy has also been demonstrated with antibody drug conjugates and a granzyme-B Fc fusion protein with an Fn14-specific scFv targeting domain (23–27). The second strategy to target the TWEAK-Fn14 system for tumor therapy is to block TWEAK-induced Fn14 activation to interfere with Fn14-mediated protumoral activities. This approach was successful in some murine tumor models (14, 28).

The third approach is the use of membrane TWEAK-mimicking agonists to unleash the full range of proinflammatory Fn14 activities. The rational basis for this approach is that membrane TWEAK, which potently activates the classical NFκB pathway, is very efficiently processed by furine proteases to release the by far less proinflammatory soluble form of TWEAK, which only poorly activates the classical NFκB pathway. Therefore, it has to be assumed that sTWEAK is *in vivo* the dominant form of TWEAK resulting in low inflammatory activity of Fn14. Treatment with a memTWEAK-mimicking Fn14 agonist should therefore induce a comprehensive and supraphysiological strong inflammatory Fn14 response with an antitumoral net effect. Indeed, as already discussed above, there is evidence that the agonism of FcγR-bound anti-Fn14 antibodies contributes to the antitumoral effects of ADCC-stimulating anti-Fn14 antibodies. Another important antitumoral effect of membrane but also soluble TWEAK-mimicking agonists is potentially the depletion of cytoplasmic TRAF2 pools and sensitization for TNF-induced cell death (4, 5). In this respect, two independent groundbreaking studies give strong evidence that tumor cell-expressed TRAF2 antagonizes the efficacy of checkpoint inhibitor therapies by protection against cell death induction by CD8^+^-derived TNF (29, 30). In sum, comprehensive and selective Fn14 engagement appears as a very interesting and promising avenue to treat cancer and could also be of possible value to exploit the Fn14-mediated regenerative responses after muscle, pancreas or liver injury (7–9). However, there are yet no Fn14 antibodies available with strong and FcγR-independent, thus purely Fn14-specific agonistic activity.

In this study, we identified oligovalent variants of the Fn14-specific antibody 18D1 which mimic the activity of sTWEAK or memTWEAK independent from FcγR binding (**Figures 2**, **5**) and which have good antitumoral activity (**Figure 6**). In contrast to conventional anti-Fn14 antibodies, the agonism of these novel Fn14 agonists is not limited by the availability of FcγR-expressing immune cells or competition with endogenous irrelevant antibodies for FcγR binding. Unexpectedly, although all oligovalent anti-Fn14 variants efficiently induced p100 processing and enhancement of TNF-induced cell death, there were considerable differences in the induction of IL8, a target of the classical NFκB pathway. Constructs only harboring multiple copies of the scFv:18D1 domain largely failed to activate IL8 production while constructs with similar valency but composed of Fab and scFv domains did this very efficiently (**Figure 2C**). Thus, the number and type of the Fn14 binding domains within an oligovalent 18D1 construct seem to decide whether sTWEAK- or memTWEAK-like activity is mimicked. The pathway selective agonism mirrors the activities of sTWEAK and memTWEAK, the natural ligands of Fn14, but is yet without precedence for anti-Fn14 antibodies. Formation of trimeric Fn14 complexes induced by sTWEAK enables the recruitment of TRAF2 and thus reduces its availability for the inhibition of the alternative NFκB pathway and TNF-induced cell killing but this is not sufficient to ensure activation of the classical NFκB pathway. However, the latter can be achieved by membrane TWEAK and FcγR-bound anti-Fn14 antibodies (15, 17, 31). Studies on Fn14-related receptors of the TNFRSF suggest furthermore that membrane-bound ligand molecules and FcγR-bound antibodies have a superior ability compared to soluble ligands and free antibodies to promote secondary clustering of initially formed trimeric TNF receptor complexes (32). It is therefore tempting to speculate that the differential classical NFκB pathway agonism of Fab/scFv domain versus scFv-only domain anti-Fn14 variants reflects a different ability to trigger supramolecular clustering. However, this remained to be clarified in future more detailed studies on the molecular mode of action of the novel Fn14 agonists, we have identified in this study.

## Materials and methods

### Cell lines, reagents and statistics

HEK293T and HT1080 cells were from the American Type Culture Collection (ATCC) (Rockville, MD, USA) or the German Collection of Microorganisms and Cell Cultures (DSMZ) (Braunschweig, Germany). HeLa-RIPK3-FADD_KO_ cells were described elsewhere (33). All cell lines were cultivated in RPMI 1640 medium (Thermo Fischer Scientific, GB, #21875-034) supplemented with 10% fetal bovine serum (FBS) (Thermo Fischer Scientific, GB, #10270-106). Expression plasmids encoding the heavy and light chains of the various recombinant proteins (**Supplemental Data Table SI**) were produced by standard cloning techniques into pCR3 (Invitrogen, Germany). Antibodies used in the study were purchased from following suppliers: Abcam (anti-Fn14 EPR3179, # ab109365), Sigma-Aldrich, Germany (anti-GAPDH 71.1, #G9295; anti-FLAG® M2, #F3165; anti-ß-actin AC-15, #A1978; anti-NFκB p52, #05-361), Cell Signaling, GB (anti-IκBα L35A5, #4818S; anti-phospho-IκBα (Ser32) 14D4, #28592; anti-TRAF-1 45D3, 70745), LI-COR Biosciences, Lincoln, USA (IRDye® 800CW anti-mouse IgG, #926-32210), Dako, Glostrup, Denmark (rabbit anti-mouse IgG with horseradish peroxidase (HRP) #P0260, goat anti-rabbit IgG with HRP, #P0448). Production and properties of the soluble TWEAK variants Flag-sTWEAK (TWEAK) and Fc-Flag-sTWEAK (Fc-TWEAK) has been described before (17) and TNF was a kind gift from Prof. Daniela Männel (University of Regensburg, Germany). Statistical analyses were performed with the corresponding functions of the GraphPad Prism software.

### Production and purification of recombinant proteins

HEK293T cells were transiently transfected with expression plasmids (ratio 1:1) encoding the heavy and light chain variants of the antibody variant of interest (**Supplemental Data Table SII**) using polyethylenimine (PEI, Polyscience Inc., Warrington, USA, #23966) essentially as described elsewhere (34). One day after adding the medium containing PEI/DNA mixture, the latter was replaced by RPMI 1640 medium supplemented with 2% FBS containing 100 U/ml penicillin and 100 µg/ml streptomycin (Sigma-Aldrich, Germany, #P4333). After additional 5-7 days supernatants were collected, cleared by centrifugation (10 min, 4600 x g) and initially assayed for the presence of recombinant proteins by western blot detection (primary antibody: anti-FLAG M2; secondary antibody anti-murine IgG IRDye 800CW). The concentration of the Flag-tagged proteins in the supernatants (SN) were estimated by anti-Flag western blot and comparison of the band intensities in the SN samples and purified Flag-tagged antibodies of known concentration used as standard. The various antibody variants were purified by anti-Flag affinity chromatography as described elsewhere (34). Concentrations and purity of purified proteins were analyzed by SDS-PAGE and silver staining of the protein gel with the Pierce Silver Stain Kit (Thermo Fischer Scientific, USA) and comparison with the protein standards of the LMW Calibration Kit for SDS Electrophoresis from Amersham (GE Healthcare). The purity and integrity of purified recombinant antibodies were furthermore analyzed using High Performance Liquid Chromatography (HPLC) (UltiMate 3000, Thermo Fischer Scientific, USA) using a MabPac SEC-1 column (Thermo Fisher, #088460).

### Western blot analysis

HT1080 cells were seeded in 12-well cell culture plates (Greiner Bio-One, Germany, #665180) (2 x 10^5^ cells/well). Next day, medium was replaced by fresh medium supplemented with the antibodies of interest. As a positive control cells were challenged with 200 ng/ml Flag-TWEAK. After 20-24 h, total cell lysates were prepared by suspending the cell pellet in Laemmli sample buffer containing, if not otherwise stated, 5% β-mercaptoethanol, sonification for 25 seconds and heating for 5 minutes at 95°C. Lysates were separated by SDS-PAGE and proteins were transferred to nitrocellulose for western blot analysis of p100 to p52 processing (primary antibody: anti-NFκB p52; secondary antibody: HRP-labeled goat anti-mouse IgG), TRAF1 induction (primary antibody: rabbit anti-TRAF1; secondary antibody: HRP-labeled goat anti-rabbit IgG) and IκBα phosphorylation (primary antibody: rabbit anti-IκBα; secondary antibody: HRP-labeled goat anti-rabbit IgG). Protein loads were controlled by detection of β-actin (primary antibody: anti-ß-actin; secondary antibody: HRP-labeled anti-mouse IgG). To analyze Fn14 expression in murine tumor organoids (MTOs), MTO lysates were separated by SDS-PAGE and transferred to nitrocellulose as described above and analyzed for Fn14 (primary antibody: rabbit anti-Fn14; secondary antibody: HRP-labeled goat anti-rabbit IgG) and GAPDH (peroxidase-labeled mouse monoclonal antibody) expression.

### Analysis of IL8 induction

HT1080 cells were seeded in 96-well cell culture plates (Sarstedt, Germany, #83.3924) (2 x 10^4^ cells/well) and the next day cells were challenged with reagents of interest for an additional 24 h. As a positive control, cells were stimulated with anti-Flag antibody M2 oligomerized Flag-TWEAK which mimics the activity of membrane TWEAK (17). Cell culture supernatants were collected and analyzed with respect to their IL8 content using the human IL8 ELISA Kit (BD Biosciences, Heidelberg, Germany, #555244).

### Enhancement of TNF-induced cell death

To analyze the ability of the various Fn14-specific antibody variants to enhance TNF-induced toxicity, HeLa-RIPK3-FADD_KO_ cells were seeded in the 96-well cell culture plates (Sarstedt) (2,5 x 10^4^ cells/well). The next day, the medium was replaced by fresh medium containing the Fn14-specific reagents of interest with or without 1 ng/ml of TNF (Männel, University of Regensburg, Germany). The next day, cell viability was finally analyzed by crystal violet staining. Viability values were normalized according to untreated cells (100% viability) and cells incubated with a toxic mixture of reagents (0% viability).

### Animal experiments

Male C57Bl/6J mice (6 weeks) were purchased from Janvier Labs (Le Genest-Saint-Isle, France) and housed at a controlled temperature of 21 ± 1°C with a dark/light cycle of 12h/12h. Mice had access to a standard chow diet and water *ad libitum*. After an adjustment period of two weeks, 5 x 10^5^ MTOs cultured as described by (18) were injected subcutaneously in the flank region, and grown over two weeks to palpable tumors. Subsequently, tumor-bearing mice received 3 intraperitoneal injections per week of MSA-muTWEAK, Fc-muTWEAK, 18D1-(2) or 18D1-(4) (all 200 µg) or NaCl (0.9%) physiological solution as a control treatment for two weeks. All animal experiments were approved by the Regierung von Unterfranken (license number AZ 2-1442) and complied with the German animal protection law.

## Data availability statement

The original contributions presented in the study are included in the article/**Supplementary Material**. Further inquiries can be directed to the corresponding author.

## Ethics statement

The animal study was reviewed and approved by Regierung von Unterfranken.

## Author contributions

OZ, ML, MA, TZ and KK produced and purified the various proteins and performed the *in vitro* assays. OZ and AH performed the *in vivo* experiments. OZ, AH, AW, CO and HW analyzed the data and wrote the manuscript. AW advised on the animal experiments. All authors contributed to the article and approved the submitted version.
