# Erratum: Antibody-based soluble and membrane-bound TWEAK mimicking agonists with FcγR-independent activity

**DOI:** 10.3389/fimmu.2023.1353758

**Published:** 2023-12-15

**Authors:** Frontiers Production Office

**Affiliations:** Frontiers Media SA, Lausanne, Switzerland

**Keywords:** agonistic antibodies, cell death, FcγR, Fn14, NFκB, TNF receptor superfamily, TWEAK

Due to a production error, there was an error regarding the affiliations for Mohamed A. Anany. As well as having affiliation 1, they should also have the affiliation “Department of Microbial Biotechnology, Institute of Biotechnology, National Research Center, Giza, Egypt”. The publisher apologizes for this mistake.

The original version of this article has been updated.

