# Supplementary material for: Antibody-based soluble and membrane-bound TWEAK mimicking agonists with FcγR-independent activity
Source: Front Immunol. 2023 Jul 11;14:1194610. doi: 10.3389/fimmu.2023.1194610 (PMC10402896; doi:10.3389/fimmu.2023.1194610)
Supplement: Supplementary file 1 [file DataSheet_1.pdf]

Supplementary Material

Antibody-based soluble and membrane-bound TWEAK mimicking agonists with FcγR-independent activity

Olena Zaitseva[1,#], Annett Hoffmann[2,#], Margareta Loest[1], Mohamed A. Anany[1], Tengyu Zhang[1], Kirstin Kucka[1], Armin Wiegering[2], Christoph Otto[2] and Harald Wajant[1,*]

[1] University Hospital Würzburg, Division of Molecular Internal Medicine, Department of Internal Medicine II, Würzburg, Germany.

[2] University Hospital Würzburg, Department of General, Visceral, Transplant, Vascular and Pediatric Surgery, Oberdürrbacher Straße 6, 97080 Würzburg, Germany.

[#] These authors equally contributed to this work.

* Corresponding author: harald.wajant@mail.uni-wuerzburg.de

Supplementary Figures

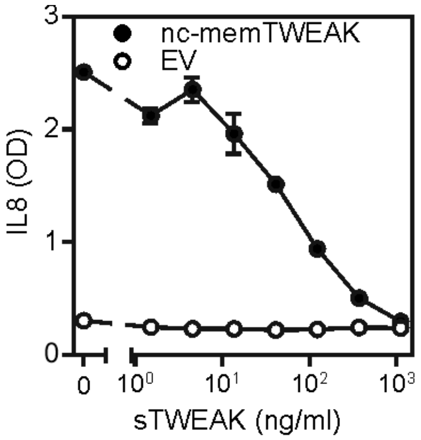

Supplemental Figure S1. HT1080 cells, which produce IL8 in response to memTWEAK expressing cells, were mixed with HEK293 cells transiently transfected with empty vector (EV, negative control) or an expression plasmid encoding a non-cleavable form of membrane TWEAK (nc-memTWEAK). Cell mixtures were further supplemented with the indicated concentration of soluble TWEAK (sTWEAK). Next day, IL8 production was evaluated by ELISA.

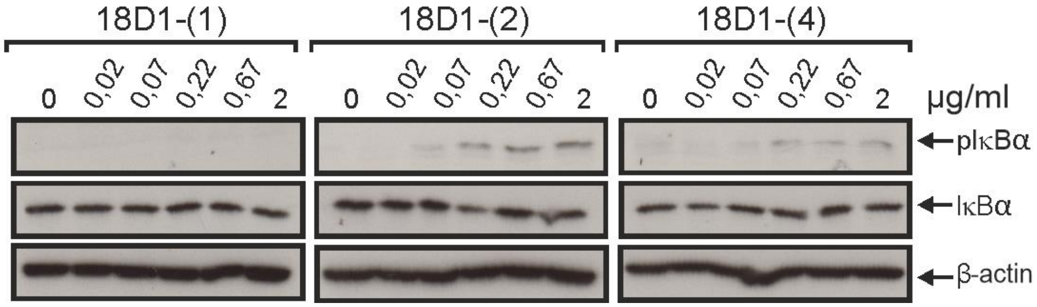

Supplemental Figure S2. The tetra- and hexavalent 18D1 variants (2) and (4) but not the parental bivalent antibody (18D1-(1)) trigger IκBα phosphorylation in HT1080 cells. Cells were stimulated with the indicated concentrations of 18D1-(1), 18D1-(2) and 18D1-(4) overnight and total lysates were finally analyzed by western blotting for the indicated proteins. Please note, at early time points the initial unbalance of the coupled processes of IκBα phosphorylation, IκBα degradation and NFκB-induced IκBα resynthesis does not allow a fair comparison of dose-responses relationships at one defined time point. We therefore analyzed a later time point when these processes have reached an equilibrium resulting in IκBα phosphorylation at constant IκBα levels.

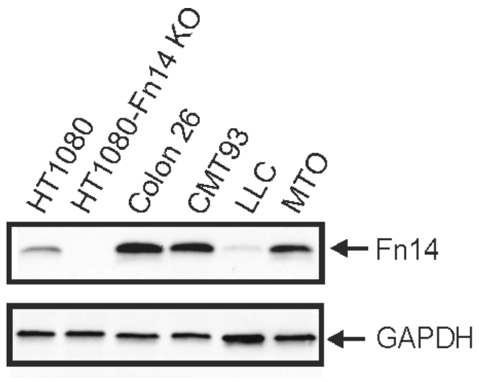

Supplemental Figure S3. Fn14 expression in murine tumor organoids (MTO) derived of tumors developing from *Apc*$^{ko/ko}$, *Kras*$^{LSL\text{-}G12D}$, *Tgfbr2*$^{ko/ko}$ and *Trp53*$^{ko/ko}$ intestinal stem cells. Total lysates of MTOs, the murine tumor cell lines Colon 26, CMT93 and Lewis lung carcinoma along with lysates of HT1080 and Fn14-deficient HT1080 cells were analyzed by western blot for the presence of Fn14, GAPDH expression was analyzed as load control.

Supplementary Tables

Supplemental table SI. AA sequences of heavy and light chain variants of Fn14-specific antibody constructs. Leader, underlined; Linker sequences, bold; Flag tag, underlined + grey background; variable domains, italic; constant IgG1 domains, grey background; TNC trimerization domain, italic + underlined + grey background.

No.	Plasmid name	Sequence
1	18D1-HC(N297A)	MNFGFSLIFLVLVLKGVQCEVKLVPRQLDYKDDDDKEFEVQLVESGGGLVQPG<br>GSLRLSCAASGFTFSNYWMSWVRQAPGKGLEWVSGINPGGTSTYYADSVKGRF<br>TISRDNAKNTLYLQMNSLKSEDTAVYYCAKHLGNWGEYNYWGQGTQVTVSSGS<br>SSASTKGPSVFPLAPSSKSTSGGTAALGCLVKDYFPEPVTVSWNSGALTSGVH<br>TFPAVLQSSGLYSLSSVVTVPSSSLGTQTYICNVNHKPSNTKVDKKVEPKSCD<br>KTHTCPPCPAPELLGGPSVFLFPPKPKDTLMISRTPEVTCVVVDVSHEDPEVK<br>FNWYVDGVEVHNAKTKPREEQYASTYRVVSVLTVLHQDWLNGKEYKCKVSNKA<br>LPAPIEKTISKAKGQPREPQVYTLPPSRDELTKNQVSLTCLVKGFYPSDIAVE<br>WESNGQPENNYKTTPPVLDSDGSFFLYSKLTVDKSRWQQGNVFSCSVMHEALH<br>NHYTQKSLSLSPGK
2	18D1-HC(N297A)-scFv:18D1	MNFGFSLIFLVLVLKGVQCEVKLVPRQLDYKDDDDKEFEVQLVESGGGLVQPG<br>GSLRLSCAASGFTFSNYWMSWVRQAPGKGLEWVSGINPGGTSTYYADSVKGRF<br>TISRDNAKNTLYLQMNSLKSEDTAVYYCAKHLGNWGEYNYWGQGTQVTVSSGS<br>SSASTKGPSVFPLAPSSKSTSGGTAALGCLVKDYFPEPVTVSWNSGALTSGVH<br>TFPAVLQSSGLYSLSSVVTVPSSSLGTQTYICNVNHKPSNTKVDKKVEPKSCD<br>KTHTCPPCPAPELLGGPSVFLFPPKPKDTLMISRTPEVTCVVVDVSHEDPEVK<br>FNWYVDGVEVHNAKTKPREEQYASTYRVVSVLTVLHQDWLNGKEYKCKVSNKA<br>LPAPIEKTISKAKGQPREPQVYTLPPSRDELTKNQVSLTCLVKGFYPSDIAVE<br>WESNGQPENNYKTTPPVLDSDGSFFLYSKLTVDKSRWQQGNVFSCSVMHEALH<br>NHYTQKSLSLSPGKLEEVQLVESGGGLVQPGGSLRLSCAASGFTFSNYWMSWV<br>RQAPGKGLEWVSGINPGGTSTYYADSVKGRFTISRDNAKNTLYLQMNSLKSED<br>TAVYYCAKHLGNWGEYNYWGQGTQVTVSSRSSTKGPKLEEGEFSEAQLQSALT<br>QPPSVSGSPGKTVTISCAGTGGDVGYRNSVSWYQQLPGMAPKLLIYDVDKRAS<br>GITDRFSGSKSGDTASLTISGVQSEDEADYYCASQRSGIAAVFGGGTHLTVLG
3	18D1-LC	MNFGFSLIFLVLVLKGVQCEVKLVPRQLDYKDDDDKEFQSALTQPPSVSGSPG<br>KTVTISCAGTGGDVGYRNSVSWYQQLPGMAPKLLIYDVDKRASGITDRFSGSK<br>SGDTASLTISGVQSEDEADYYCASQRSGIAAVFGGGTHLTVLGGSEIKRTVAA<br>PSVFIFPPSDEQLKSGTASVVCLLNNFYPREAKVQWKVDNALQSGNSQESVTE<br>QDSKDSTYSLSSTLTLSKADYEKHKVYACEVTHQGLSSPVTKSFNRGEC
4	18D1-LC-scFv:18D1	MNFGFSLIFLVLVLKGVQCEVKLVPRQLDYKDDDDKEFQSALTQPPSVSGSPG<br>KTVTISCAGTGGDVGYRNSVSWYQQLPGMAPKLLIYDVDKRASGITDRFSGSK<br>SGDTASLTISGVQSEDEADYYCASQRSGIAAVFGGGTHLTVLGGSEIKRTVAA<br>PSVFIFPPSDEQLKSGTASVVCLLNNFYPREAKVQWKVDNALQSGNSQESVTE<br>QDSKDSTYSLSSTLTLSKADYEKHKVYACEVTHQGLSSPVTKSFNRGECLEEV<br>QLVESGGGLVQPGGSLRLSCAASGFTFSNYWMSWVRQAPGKGLEWVSGINPGG<br>TSTYYADSVKGRFTISRDNAKNTLYLQMNSLKSEDTAVYYCAKHLGNWGEYNY<br>WGQGTQVTVSSRSSTKGPKLEEGEFSEAQLQSALTQPPSVSGSPGKTVTISCA<br>GTGGDVGYRNSVSWYQQLPGMAPKLLIYDVDKRASGITDRFSGSKSGDTASLT<br>ISGVQSEDEADYYCASQRSGIAAVFGGGTHLTVLG
5	scFv:18D1-CL	MNFGFSLIFLVLVLKGVQCEVKLVPRQLDYKDDDDKEFEVQLVESGGGLVQPG<br>GSLRLSCAASGFTFSNYWMSWVRQAPGKGLEWVSGINPGGTSTYYADSVKGRF<br>TISRDNAKNTLYLQMNSLKSEDTAVYYCAKHLGNWGEYNYWGQGTQVTVSSRS<br>STKGPKLEEGEFSEAQLQSALTQPPSVSGSPGKTVTISCAGTGGDVGYRNSVS<br>WYQQLPGMAPKLLIYDVDKRASGITDRFSGSKSGDTASLTISGVQSEDEADYY<br>CASQRSGIAAVFGGGTHLTVLGGSEIKRTVAAPSVFIFPPSDEQLKSGTASVV<br>CLLNNFYPREAKVQWKVDNALQSGNSQESVTEQDSKDSTYSLSSTLTLSKADY<br>EKHKVYACEVTHQGLSSPVTKSFNRGEC

6	scFv:18D1-CH1,2,3(N297A)	MNFGFSLIFLVLVLKGVQCEVKLVPRQLDYKDDDDKEFEVQLVESGGGLVQPG<br>GSLRLSCAASGFTFSNYWMSWVRQAPGKGLEWVSGINPGGTSTYYADSVKGRF<br>TISRDNAKNTLYLQMNSLKSEDTAVYYCAKHLGNWGEYNYWGQGTQVTVSSRS<br>STKGPKLEEGEFSEAQLQSALTQPPSVSGSPGKTVTISCAGTGGDVGYRNSVS<br>WYQQLPGMAPKLLIYDVDKRASGITDRFSGSKSGDTASLTISGVQSEDEADYY<br>CASQRSGIAAVFGGGTHLTVLGGSSSASTKGPSVFPLAPSSKSTSGGTAALGC<br>LVKDYFPEPVTVSWNSGALTSGVHTFPAVLQSSGLYSLSSVVTVPSSSLGTQT<br>YICNVNHKPSNTKVDKKVEPKSCDKTHTCPPCPAPELLGGPSVFLFPPKPKDT<br>LMISRTPEVTCVVVDVSHEDPEVKFNWYVDGVEVHNAKTKPREEQYASTYRVV<br>SVLTVLHQDWLNGKEYKCKVSNKALPAPIEKTISKAKGQPREPQVYTLPPSRD<br>ELTKNQVSLTCLVKGFYPSDIAVEWESNGQPENNYKTTPPVLDSDGSFFLYSK<br>LTVDKSRWQQGNVFSCSVMHEALHNHYTQKSLSLSPGK
7	scFv:18D1-CL-scFv:18D1	MNFGFSLIFLVLVLKGVQCEVKLVPRQLDYKDDDDKEFEVQLVESGGGLVQPG<br>GSLRLSCAASGFTFSNYWMSWVRQAPGKGLEWVSGINPGGTSTYYADSVKGRF<br>TISRDNAKNTLYLQMNSLKSEDTAVYYCAKHLGNWGEYNYWGQGTQVTVSSRS<br>STKGPKLEEGEFSEAQLQSALTQPPSVSGSPGKTVTISCAGTGGDVGYRNSVS<br>WYQQLPGMAPKLLIYDVDKRASGITDRFSGSKSGDTASLTISGVQSEDEADYY<br>CASQRSGIAAVFGGGTHLTVLGGSEIKRTVAAPSVFIFPPSDEQLKSGTASVV<br>CLLNNFYPREAKVQWKVDNALQSGNSQESVTEQDSKDSTYSLSSTLTLSKADY<br>EKHKVYACEVTHQGLSSPVTKSFNRGECLEEVQLVESGGGLVQPGGSLRLSCA<br>ASGFTFSNYWMSWVRQAPGKGLEWVSGINPGGTSTYYADSVKGRFTISRDNAK<br>NTLYLQMNSLKSEDTAVYYCAKHLGNWGEYNYWGQGTQVTVSSRSSTKGPKLE<br>EGEFSEAQLQSALTQPPSVSGSPGKTVTISCAGTGGDVGYRNSVSWYQQLPGM<br>APKLLIYDVDKRASGITDRFSGSKSGDTASLTISGVQSEDEADYYCASQRSGI<br>AAVFGGGTHLTVLG
8	scFv:18D1-CH1,2,3(N297A)-scFv:18D1	MNFGFSLIFLVLVLKGVQCEVKLVPRQLDYKDDDDKEFEVQLVESGGGLVQPG<br>GSLRLSCAASGFTFSNYWMSWVRQAPGKGLEWVSGINPGGTSTYYADSVKGRF<br>TISRDNAKNTLYLQMNSLKSEDTAVYYCAKHLGNWGEYNYWGQGTQVTVSSRS<br>STKGPKLEEGEFSEAQLQSALTQPPSVSGSPGKTVTISCAGTGGDVGYRNSVS<br>WYQQLPGMAPKLLIYDVDKRASGITDRFSGSKSGDTASLTISGVQSEDEADYY<br>CASQRSGIAAVFGGGTHLTVLGGSSSASTKGPSVFPLAPSSKSTSGGTAALGC<br>LVKDYFPEPVTVSWNSGALTSGVHTFPAVLQSSGLYSLSSVVTVPSSSLGTQT<br>YICNVNHKPSNTKVDKKVEPKSCDKTHTCPPCPAPELLGGPSVFLFPPKPKDT<br>LMISRTPEVTCVVVDVSHEDPEVKFNWYVDGVEVHNAKTKPREEQYASTYRVV<br>SVLTVLHQDWLNGKEYKCKVSNKALPAPIEKTISKAKGQPREPQVYTLPPSRD<br>ELTKNQVSLTCLVKGFYPSDIAVEWESNGQPENNYKTTPPVLDSDGSFFLYSK<br>LTVDKSRWQQGNVFSCSVMHEALHNHYTQKSLSLSPGKLEEVQLVESGGGLVQ<br>PGGSLRLSCAASGFTFSNYWMSWVRQAPGKGLEWVSGINPGGTSTYYADSVKG<br>RFTISRDNAKNTLYLQMNSLKSEDTAVYYCAKHLGNWGEYNYWGQGTQVTVSS<br>RSSTKGPKLEEGEFSEAQLQSALTQPPSVSGSPGKTVTISCAGTGGDVGYRNS<br>VSWYQQLPGMAPKLLIYDVDKRASGITDRFSGSKSGDTASLTISGVQSEDEAD<br>YYCASQRSGIAAVFGGGTHLTVLG
9	PDL192-HC(N297A)	MNFGFSLIFLVLVLKGVQCEVKLVPRQLDYKDDDDKEFEVQLVESGGGLVQPG<br>GSLRLSCAASGFTFSSYWMSWVRQAPGKGLEWVAEIRLKSDNYATHYAESVKG<br>RFTISRDDSKNSLYLQMNSLRAEDTAVYYCTGYYADAMDYWGQGTLVTVSSGS<br>SSASTKGPSVFPLAPSSKSTSGGTAALGCLVKDYFPEPVTVSWNSGALTSGVH<br>TFPAVLQSSGLYSLSSVVTVPSSSLGTQTYICNVNHKPSNTKVDKKVEPKSCD<br>KTHTCPPCPAPELLGGPSVFLFPPKPKDTLMISRTPEVTCVVVDVSHEDPEVK<br>FNWYVDGVEVHNAKTKPREEQYASTYRVVSVLTVLHQDWLNGKEYKCKVSNKA<br>LPAPIEKTISKAKGQPREPQVYTLPPSRDELTKNQVSLTCLVKGFYPSDIAVE<br>WESNGQPENNYKTTPPVLDSDGSFFLYSKLTVDKSRWQQGNVFSCSVMHEALH<br>NHYTQKSLSLSPGK
10	PDL192-HC(N297A)-scFv:PDL192	MNFGFSLIFLVLVLKGVQCEVKLVPRQLDYKDDDDKEFEVQLVESGGGLVQPG<br>GSLRLSCAASGFTFSSYWMSWVRQAPGKGLEWVAEIRLKSDNYATHYAESVKG<br>RFTISRDDSKNSLYLQMNSLRAEDTAVYYCTGYYADAMDYWGQGTLVTVSSGS<br>SSASTKGPSVFPLAPSSKSTSGGTAALGCLVKDYFPEPVTVSWNSGALTSGVH<br>TFPAVLQSSGLYSLSSVVTVPSSSLGTQTYICNVNHKPSNTKVDKKVEPKSCD<br>KTHTCPPCPAPELLGGPSVFLFPPKPKDTLMISRTPEVTCVVVDVSHEDPEVK<br>FNWYVDGVEVHNAKTKPREEQYASTYRVVSVLTVLHQDWLNGKEYKCKVSNKA<br>LPAPIEKTISKAKGQPREPQVYTLPPSRDELTKNQVSLTCLVKGFYPSDIAVE<br>WESNGQPENNYKTTPPVLDSDGSFFLYSKLTVDKSRWQQGNVFSCSVMHEALH<br>NHYTQKSLSLSPGKEFEVQLVESGGGLVQPGGSLRLSCAASGFTFSSYWMSWV<br>RQAPGKGLEWVAEIRLKSDNYATHYAESVKGRFTISRDDSKNSLYLQMNSLRA<br>EDTAVYYCTGYYADAMDYWGQGTLVTVSSRSSTKGPKLEEGEFSEAQLDIQMT

		QSPSSLSASVGDRVTITCRASQSVSTSSYSYMHWYQQKPGKAPKLLIKYASNL<br>ESGVPSRFSGSGSGTDFTLTISSLQPEDFATYYCQHSWEIPYTFGGGTKVEIK
11	PDL192-LC	MNFGFSLIFLVLVLKGVQCEVKLVPRQLDYKDDDDKEFDIQMTQSPSSLSASV<br>GDRVTITCRASQSVSTSSYSYMHWYQQKPGKAPKLLIKYASNLESGVPSRFSG<br>SGSGTDFTLTISSLQPEDFATYYCQHSWEIPYTFGGGTKVEIKRTVAAPSVFI<br>FPPSDEQLKSGTASVVCLLNNFYPREAKVQWKVDNALQSGNSQESVTEQDSKD<br>STYSLSSTLTLSKADYEKHKVYACEVTHQGLSSPVTKSFNRGEC
12	PDL192-LC-scFv:PDL192	MNFGFSLIFLVLVLKGVQCEVKLVPRQLDYKDDDDKEFDIQMTQSPSSLSASV<br>GDRVTITCRASQSVSTSSYSYMHWYQQKPGKAPKLLIKYASNLESGVPSRFSG<br>SGSGTDFTLTISSLQPEDFATYYCQHSWEIPYTFGGGTKVEIKRTVAAPSVFI<br>FPPSDEQLKSGTASVVCLLNNFYPREAKVQWKVDNALQSGNSQESVTEQDSKD<br>STYSLSSTLTLSKADYEKHKVYACEVTHQGLSSPVTKSFNRGECEFEVQLVES<br>GGGLVQPGGSLRLSCAASGFTFSSYWMSWVRQAPGKGLEWVAEIRLKSDNYAT<br>HYAESVKGRFTISRDDSKNSLYLQMNSLRAEDTAVYYCTGYYADAMDYWGQGT<br>LVTVSSRSSTKGPKLEEGEFSEAQLDIQMTQSPSSLSASVGDRVTITCRASQS<br>VSTSSYSYMHWYQQKPGKAPKLLIKYASNLESGVPSRFSGSGSGTDFTLTISS<br>LQPEDFATYYCQHSWEIPYTFGGGTKVEIK
13	scFv:PDL192-CL	MNFGFSLIFLVLVLKGVQCEVKLVPRQLDYKDDDDKEFEVQLVESGGGLVQPG<br>GSLRLSCAASGFTFSSYWMSWVRQAPGKGLEWVAEIRLKSDNYATHYAESVKG<br>RFTISRDDSKNSLYLQMNSLRAEDTAVYYCTGYYADAMDYWGQGTLVTVSSRS<br>STKGPKLEEGEFSEAQLDIQMTQSPSSLSASVGDRVTITCRASQSVSTSSYSY<br>MHWYQQKPGKAPKLLIKYASNLESGVPSRFSGSGSGTDFTLTISSLQPEDFAT<br>YYCQHSWEIPYTFGGGTKVEIKGSEIKRTVAAPSVFIFPPSDEQLKSGTASVV<br>CLLNNFYPREAKVQWKVDNALQSGNSQESVTEQDSKDSTYSLSSTLTLSKADY<br>EKHKVYACEVTHQGLSSPVTKSFNRGEC
14	scFv:PDL192-<br>$CH_{1,2,3}$(N297A)	MNFGFSLIFLVLVLKGVQCEVKLVPRQLDYKDDDDKEFEVQLVESGGGLVQPG<br>GSLRLSCAASGFTFSSYWMSWVRQAPGKGLEWVAEIRLKSDNYATHYAESVKG<br>RFTISRDDSKNSLYLQMNSLRAEDTAVYYCTGYYADAMDYWGQGTLVTVSSRS<br>STKGPKLEEGEFSEAQLDIQMTQSPSSLSASVGDRVTITCRASQSVSTSSYSY<br>MHWYQQKPGKAPKLLIKYASNLESGVPSRFSGSGSGTDFTLTISSLQPEDFAT<br>YYCQHSWEIPYTFGGGTKVEIKGSSSASTKGPSVFPLAPSSKSTSGGTAALGC<br>LVKDYFPEPVTVSWNSGALTSGVHTFPAVLQSSGLYSLSSVVTVPSSSLGTQT<br>YICNVNHKPSNTKVDKKVEPKSCDKTHTCPPCPAPELLGGPSVFLFPPKPKDT<br>LMISRTPEVTCVVVDVSHEDPEVKFNWYVDGVEVHNAKTKPREEQYASTYRVV<br>SVLTVLHQDWLNGKEYKCKVSNKALPAPIEKTISKAKGQPREPQVYTLPPSRD<br>ELTKNQVSLTCLVKGFYPSDIAVEWESNGQPENNYKTTPPVLDSDGSFFLYSK<br>LTVDKSRWQQGNVFSCSVMHEALHNHYTQKSLSLSPGK

Supplemental table SII. Heavy and light chain composition of Fn14-specific antibody variants.

**Antibody variant**	**Plasmid no. from supplemental table I**
18D1-(1)	1+3
18D1-(2)	2+3
18D1-(3)	1+4
18D1-(4)	2+4
18D1-(5)	6+5
18D1-(6)	8+5
18D1-(7)	6+7
18D1-(8)	8+7
PDL192-(1)	9+11
PDL192-(2)	10+11
PDL192-(3)	9+12
PDL192-(4)	10+12
PDL192-(5)	14+13

frontiers
